# Congenital Surfactant C Deficiency with Pulmonary Hypertension—A Case Report

**DOI:** 10.3390/children9101435

**Published:** 2022-09-21

**Authors:** Wei Chard Chua, I-Chen Chen, Yi-Ching Liu, Yen-Hsien Wu, Shih-Hsing Lo, Jong-Hau Hsu, Peir-In Liang, Hsiu-Lin Chen, Zen-Kong Dai

**Affiliations:** 1 Department of Pediatrics, Kaohsiung Medical University Hospital, Kaohsiung 807, Taiwan; 2 Department of Pediatrics, School of Medicine, College of Medicine, Kaohsiung Medical University, Kaohsiung 807, Taiwan; 3 Graduate Institute of Medicine, College of Medicine, Kaohsiung Medical University, Kaohsiung 807, Taiwan; 4 Department of Pathology, Kaohsiung Medical University Hospital, Kaohsiung 807, Taiwan; 5 Department of Respiratory Therapy, College of Medicine, Kaohsiung Medical University, Kaohsiung 807, Taiwan

**Keywords:** pediatric, childhood interstitial lung disease, diffuse lung disease, genetic testing, hydroxychloroquine, pulmonary hypertension

## Abstract

Interstitial lung diseases in children are a diverse group in terms of etiology and pathogenesis. With advances in genetic testing, mutations in surfactant protein have now been identified as the etiology for childhood interstitial lung disease of variable onset and severity, ranging from fatal acute respiratory distress syndrome (RDS) in neonates to chronic lung disease in adults. We presented an 11-month-old girl with surfactant protein C deficiency and secondary pulmonary hypertension, successfully treated with hydroxychloroquine, and provided a detailed discussion of the clinical and diagnostic approach and management.

## 1. Introduction

Diffuse lung disease (DLD) in children, previously known as childhood interstitial lung disease (chILD), is an umbrella term for a heterogenous group of respiratory disorders that affect the parenchyma of the lungs and may cause acute or chronic lung disease [1]. The clinical manifestations vary from asymptomatic presentation to long-term oxygen (O_2_) dependence or mechanical ventilation [1]. DLD in children was classified based upon patient age at presentation, review of clinical, imaging, and lung biopsy data. In the past decade, different classification systems for chILD have been proposed [2]. Before, even with a thorough evaluation, it was not always possible to establish a definitive diagnosis of chILD. In recent years, owing to improved genetic tools, several surfactant-related gene mutations have been increasingly acknowledged as causes of DLD in infants [3].

Pulmonary surfactant is a complex mixture of lipids and proteins that reduce surface tension at the air–liquid interface of lung alveoli and prevent end collapse at the end of expiration [4,5,6]. Although protein components (surfactant protein A (SP-A), surfactant protein B (SP-B), surfactant protein C (SP-C), and surfactant protein D (SP-D)) only constitute approximately 10% of surfactant by weight, these have an important role in surfactant structure, function, and metabolism [4,5,6]. Genetic mutations resulting in surfactant protein dysfunction syndrome (SPDS) are a rare cause of acute respiratory distress in infants and chronic respiratory disease in older children [3,4].

Surfactant protein C deficiency (SPCD) is one of the SPDSs that can be inherited in an autosomal dominant pattern or can emerge via de novo mutations without family history [4,5,6]. SP-C deficiency has a highly variable age of onset and disease course with a more prominent trend to present later in childhood, associated with DLD in children and young adults [4,5,6].

Due to the rarity of SP-C deficiency, clinical cases and evidence-based treatment options for SP-C deficiency are scarce in the medical literature, especially in the Asian population. In this article, we present an 11-month-old female infant diagnosed with SP-C deficiency with secondary pulmonary hypertension, successfully treated with hydroxychloroquine.

## 2. Case

An 11-month-old female infant was admitted to the pediatric pulmonology department for chronic cough, intermittent fever for 2 days, cyanosis, and failure to thrive.

Tracing back to her history, she was born at term (38 weeks, 6 days) via vaginal delivery and had no neonatal respiratory distress or complications. There was no remarkable family history or congenital anomaly. The patient was first admitted to the hospital at three months of age with severe respiratory distress, and acute bronchiolitis was impressed. Since that episode, she presented with chronic productive cough and easy cyanosis while coughing, crying, or irritability.

On initial presentation to our ward, physical examination showed clubbing fingers, suprasternal retraction, bilateral crackles, and wheezing breathing sounds. Her height and weight were both significantly below that of other children of similar age and sex (<third percentile). We kept monitoring her oxygen saturation, which could mostly be maintained above 90% under an oxygen supply of 2–3 L/min if she was calm. Fluctuation of oxygen saturation (50–90%) was recorded with agitation or irritable crying.

**Figure 1:**
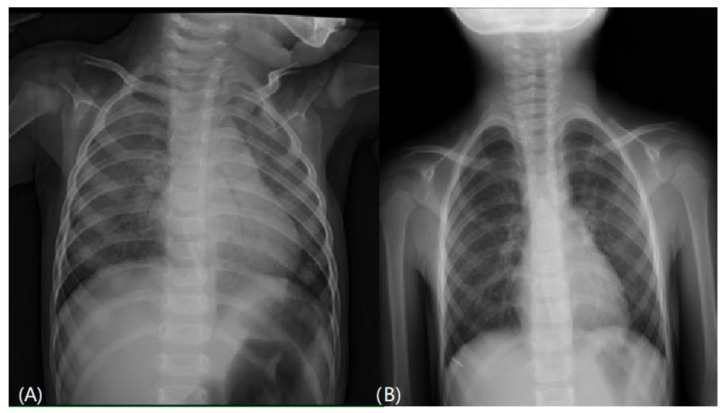
(**A**) Chest x-ray on admission showed diffuse increased interstitial density of both lungs. (**B**) Chest x-ray after treatment revealed no radiologic evidence of active cardiopulmonary disease.

**Figure 2:**
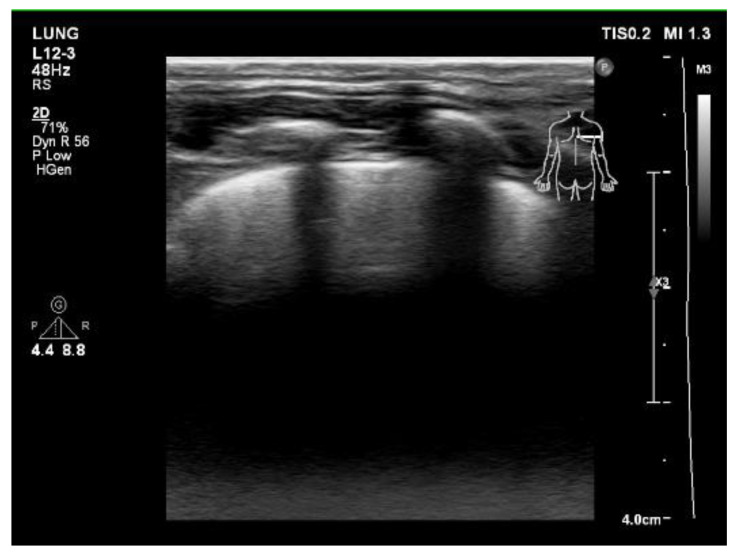
Lung echo showed multiple B lines (comet-tail artifacts), suggesting pulmonary edema.

A series of examinations was performed to establish the final diagnosis. Her blood gas pH value was within the normal range, without carbon dioxide retention or metabolic acidosis. A chest x-ray showed diffusely increased density of both lungs (Figure 1A). Lung echo suggested pulmonary edema (Figure 2), and furosemide was administered. Chest

**Figure 3:**
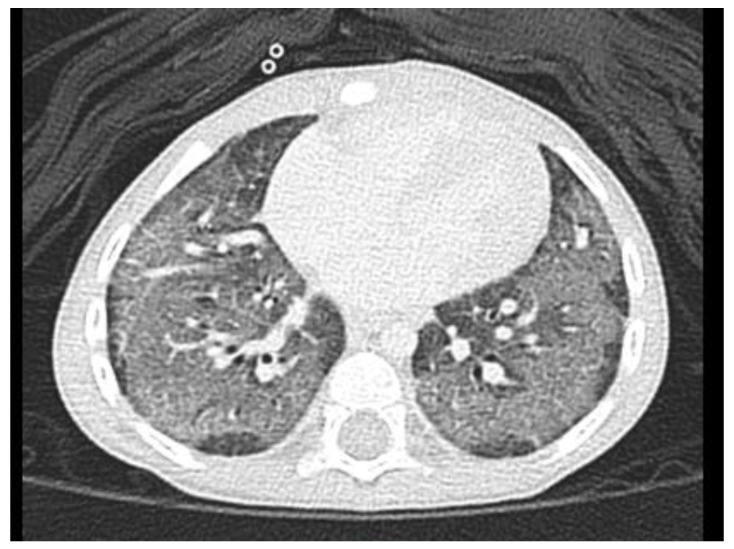
Diffuse ground-glass opacities with subpleural sparing and interstitial thickenings are noted in both lungs, with suspect interstitial pneumonia in both lungs as a crazy-paving appearance.

Computed tomography (Figure 3) showed suspected interstitial pneumonia in both lungs with a crazy-paving appearance. Bronchoscopy was performed, and no evidence of bleeding or micro-aspiration was noted in bronchoalveolar lavage (BAL) with negative culture. Pulmonary hypertension with an estimated systolic pulmonary artery pressure of 45 mmHg was revealed on echocardiography, and sildenafil treatment (1 mg/kg/day, 3 doses/day) was started. Based on the above examinations, children’s interstitial lung disease (chILD)-induced secondary pulmonary hypertension was assessed, and steroid (Betamethasone 0.04 mg/kg/day, 3 times per day) and azithromycin (10 mg/kg/day, 3 days/week) were given as anti-inflammatories. She was then discharged in relatively stable condition, with medication as described above and home care oxygen supply.

**Figure 4:**
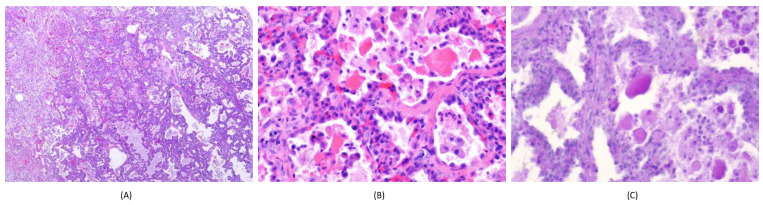
(**A**) The lung showed diffuse interstitial thickening and florid pneumocyte proliferation with increased chronic inflammatory infiltrates; (**B**) at high power, type II pneumocyte hyperplasia was seen with eosinophilic amorphous materials in the alveolar spaces; (**C**) the amorphous material was positive for PAS stain.

However, she was still hospitalized frequently because of desaturation episodes and recurrent upper airway infection. For the diagnosis of interstitial lung disease, further evaluation, including genetic analysis and lung biopsy, was suggested. Because genetic analysis in Taiwan at that time was not as popular as it is currently, and because this would have constituted a heavy economic burden for the family, open lung biopsy was performed, following discussion with the family. Histopathological examination (Figure 4) showed a thickened alveolar septum lined by reactive pneumocytes and infiltrated with chronic inflammatory cells. In the alveolar space, eosinophilic, crystal-like to amorphous material was noted, reminiscent of the eosinophilic material seen in pulmonary alveolar proteinosis (PAP). The material was PAS-positive. Based on the histological picture, diagnosis of surfactant mutation or neonatal pulmonary alveolar proteinosis was considered. Finally, for a definite genetic diagnosis, a whole exome sequence analysis was performed and the heterozygous disease-causing surfactant protein C gene (SFTPC) mutation Ile73Thr at exon 3 was found.

After establishing the final diagnosis of SP-C deficiency, we started the treatment with hydroxychloroquine (10 mg/kg/day in two divided doses). We discontinued azithromycin, and gradually tapered down steroid dosage within one month. She was then regularly followed up with through the outpatient department. After two months of treatment with hydroxychloroquine, her condition had markedly improved and her oxygen demand decreased gradually. Follow-up echocardiography was normal, without pulmonary hypertension; therefore, we discontinued sildenafil use. She took hydroxychloroquine for 21 months, and room air was well tolerated without cyanosis or exertional dyspnea. A chest X-ray revealed normal findings (Figure 1B), although lung function tests performed after halting medication still showed restrictive pulmonary disease. She was well fed after respiration improved. Her height (28th percentile) and weight (41st percentile) also caught up to the normal range. Two years after withdrawing the medication, the patient was four years old and she remained well, with no clinical or radiological signs of relapse.

## 3. Discussion

ChILD comprises a diverse group of disorders that are classified together because of similar clinical, radiographic, physiologic, or pathologic manifestations. Due to the highly variable clinical presentation, diagnosis of chILD is always a challenge [1]. Some authors had suggested investigation algorithms to diagnose child [2,7].

ChILD must be considered for the neonate who presents with unexplained respiratory failure or children suffering from persistent respiratory complaints. The first approach is to exclude the more common causes of lung disease, such as infection, asthma, immunodeficiency, structural airway abnormalities, congenital heart disease, and pulmonary hypertension [1].

High-resolution computed tomography (HRCT) is an important tool for diagnosis [1,8]. The most common HRCT-scan feature of ILD is diffuse ground-glass attenuation. The other findings included irregular interlobular septal thickening, sub-pleural reticulations, honeycombing, traction cysts, and bronchiectasis [1,8]. However, it is difficult to use HRCT alone to identify chILD. It can usually only suggest a diagnostic pattern and guide further testing. Lung biopsy and histological investigations were considered gold-standard tools for chILD diagnosis before the availability of genetic testing. Histopathologic appearance is important for chILD classification but does not necessarily provide a specific diagnosis. Currently, the few established diagnosis flowcharts of chILD suggest performing lung biopsy only if both lung images and genetic testing cannot lead to the particular diagnosis, or if disease progression is rapid and there is insufficient time for genetic testing [1,6].

Genetic tests are becoming more and more important for the diagnosis of DLD in children, with multiple disorders having an identified genetic cause [3]. Genetic testing can prevent the need for more invasive evaluations such as lung biopsy, and potentially yield a specific diagnosis, which is important for prognosis and genetic counseling for the family [6]. Thus, each child with suspected ILD should undergo genetic tests early in the diagnostic workup, prior to a lung biopsy [8], especially those with a positive family history of SPDS or unexplained respiratory symptoms [6].

SP-C is a small hydrophobic protein that stabilizes and enhances the spreading of surfactant phospholipids along the alveolar surface. It is encoded by a single gene located on chromosome 8, synthesized in pulmonary alveolar type II cells as larger precursor molecules (pro-SPC) before processing into mature forms [6]. SFTPC mutations can be inherited in an autosomal dominant pattern or can arise from de novo mutations and cause sporadic lung disease. The most common SFTPC mutation is the substitution of threonine for isoleucine in codon 73 (I73T) [6], which may account for as high as 50%of the SFTPC mutations identified so far [9]. The precise pathophysiology of the disorder is not well understood. The lung disease may be related to the gain-of-toxic-function mechanism because of the production of the mutant protein. Intracellular accumulation of abnormal misfolded pro-SPC induces an unfolded protein response, resulting in inflammation and cell apoptosis. Alternatively, the wild-type pro-SPC may be deconstructed and degraded in a dominant-negative mechanism [5,6]. SP-C deficiency has a highly variable age of onset, disease course, and severity. Some SPCD infants manifest at birth with acute respiratory distress, but the majority of patients with sequence variations in SFTPC present later in life, ranging from early infancy to well into adulthood [4,5,6].

Due to its rarity, there have been no standardized therapeutic interventions in children with SP-C deficiency. Currently, treatment of SP-C deficiency originates from the management of chILD. Pharmacological treatment with corticosteroids, hydroxychloroquine, or azithromycin has been reported to be useful in children with SPCD [1,6,10]. However, Griese et al. revealed that hydroxychloroquine had no significant treatment effect in the treatment of chILD [11]. In this case, the treatment of hydroxychloroquine seemed to be effective [1,6,10]. The exact mechanism of action of hydroxychloroquine is unknown. Besides having anti-inflammatory features, hydroxychloroquine has been shown to inhibit the intracellular processing of the precursor of SP-C and interfere with abnormal pro-SPC accumulation, which is assumed to account for its therapeutic effects [11,12].

The appropriate duration of treatment is still undetermined. A case series reported that the duration of hydroxychloroquine treatment may be important for disease prognosis [11]. In that study, the patients who had a better long-term prognosis underwent a mean duration of treatment of 25.6 months, while the patients who had residual symptoms only received treatment for 10.5 months [11]. Our patient was treated with hydroxychloroquine for 21 months. There was an apparent response to treatment. However, the follow-up lung function test still revealed restrictive lung disease without clinical evidence. Long-term follow-up, including clinical presentation, image study, and lung function tests were still needed for the patient after treatment was halted.

Pulmonary hypertension was reported as an additional comorbidity that can complicate the course of the disease and have a negative impact on prognosis [13]. A systemic review found that the estimated pulmonary hypertension in chILD diagnosed via cardiac catheterization, echocardiogram, and/or electrocardiogram ranged from 25% to 64% [13]. The European protocol and the American Thoracic Society Clinical Practice Guideline both recommended that an echocardiogram should be performed as part of the initial evaluation to recognize evidence of pulmonary hypertension earlier [7,10]. Here, we treated pulmonary hypertension symptomatically with sildenafil alongside other treatments. Pulmonary hypertension was relieved after lung condition improved.

In conclusion, in early life, chILD should be considered in patients presenting with persistent respiratory symptoms, recurrent lower respiratory tract infections, and growth retardation. Hydroxychloroquine seems to be an effective treatment for SPCD patients, but the duration of treatment remains undetermined and long-term follow-up is warranted.
